# Changes in Obesity Phenotype Distribution in Mixed-ancestry South Africans in Cape Town Between 2008/09 and 2014/16

**DOI:** 10.3389/fendo.2019.00753

**Published:** 2019-11-06

**Authors:** Saarah Fatoma Davids, Tandi Edith Matsha, Nasheeta Peer, Rajiv Timothy Erasmus, Andre Pascal Kengne

**Affiliations:** ^1^Department of Medicine, Faculty of Health Science, University of Cape Town, Cape Town, South Africa; ^2^SAMRC/CPUT/Cardiometabolic Health Research Unit, Department of Biomedical Sciences, Faculty of Health and Wellness Sciences, Cape Peninsula University of Technology, Bellville, South Africa; ^3^Non-Communicable Diseases Research Unit, South African Medical Research Council, Cape Town, South Africa; ^4^Department of Chemical Pathology, Faculty of Medicine and Health Sciences, and National Health Laboratory Service (NHLS), Stellenbosch University, Cape Town, South Africa

**Keywords:** obesity phenotype, body mass index, cardiometabolic risk factors, trends, Africa

## Abstract

**Background:** The concept of obesity phenotypes encompasses a different approach to evaluating the relationship between obesity and cardiometabolic diseases. Considering the minimal research on obesity phenotypes in Africa, we investigated these changes from 2008/09 to 2014/16 in the mixed ancestry population in Cape Town, South Africa.

**Methods:** In all, 928 (2008/09) and 1969 (2014/16) ≥20 year old participants were included in two community-based cross-sectional studies. For obesity phenotype classification, a combination of body mass index (BMI) categories and prevalent cardiometabolic disease risk factors were used, with the presence of ≥2 cardiometabolic abnormalities defining abnormal metabolic status. Interaction tests were used to investigate changes in their distribution across the years of study.

**Results:** Distribution of BMI categories differed significantly between the 2 years; normal weight, overweight and obese: 27.4, 27.4, and 45.3% in 2008/09 vs. 34.2, 23.6, and 42.2% in 2014/16 (*p* = 0.001). There was no differential effect in the distribution of obesity phenotypes pattern across the two time-points (interaction *p* = 0.126). Across BMI categories, levels of cardiometabolic risk factors linearly deteriorated in both metabolically healthy and abnormal participants (all *p* ≤ 0.018 for linear trends). Findings were not sensitive to the number of metabolic abnormalities included in the definition of obesity phenotypes.

**Conclusions:** Our study showed negligible differences in obesity phenotypes over time, but a high burden of metabolic abnormalities among normal weight participants, and a significant proportion of metabolically health obese individuals. Further investigation is needed to improve risk stratification and cost-effective identification of individuals at high risk for cardiometabolic diseases.

## Introduction

In 2011, the World Health Organization (WHO) reported that obesity had reached epidemic proportions globally, accounting for an estimated 2.8 million deaths annually (1). A further 35.8 million disability-adjusted life-years (DALYs) were attributed to overweight and obesity (1). Even in Africa, over one-quarter of adults ≥20 years of age were estimated to be overweight (2). The adoption of western lifestyles, characterized by energy-rich diets and physical inactivity, is considered to be a key contributor to the high and rising adiposity levels on the continent (3–7). South Africa is among the countries with the highest adiposity levels in Africa with a crude prevalence of 48.3 and 22.8% for overweight and obesity, respectively (8).

Despite the major influence of obesity on the development of cardiovascular diseases worldwide, the impact of obesity on cardiovascular diseases in Africa may not be uniform or comparable to other populations (9). Therefore, the concept of ‘obesity' or ‘body size' phenotype was developed (10) to evaluate the cardiometabolic risk associated with excess body weight (11–15). Unlike the metabolic syndrome, which shares common characteristics with obesity phenotype, the latter has no uniform definition and researchers use their discretion per the population being studied. For the measure of adiposity, many advocate the use of waist circumference (WC) with a focus on visceral adiposity (16, 17). However, Despres et al. (18) proposed that visceral adiposity alone was not sufficient to diagnose obesity. Therefore, body mass index (BMI) maybe more appropriately defined for the obesity phenotype concept since BMI is a stronger predictor of CVD than more accurate measures of adiposity such as percentage of body fat (19). Therefore, using BMI to define the latter, in the current study, we aim to determine the distribution and change in obesity or body size phenotypes between 2008/09 and 2014/16 in an urban South African population.

## Materials and Methods

### Study Population and Sampling Procedure

Two independent cross-sectional surveys were conducted in the mixed ancestry population of the Bellville-South Township in Cape Town in 2008/09 and 2014/16. Recruiters approached each dwelling and invited residents who fulfilled the inclusion criteria to participate in the survey. These included mixed ancestry residents aged 18 years and older (2008/09 survey) or 20 years and older (2014/16 survey) who were neither pregnant nor bed-ridden. The 2008/09 survey comprised 946 participants, as previously described (20), while the 2014/16 survey included 1989 participants. For this analysis, participants with missing data, and those who were underweight or <20 years old in the 2008/09 study, were excluded. The final sample consisted of 927 in 2008/09 and 1969 in 2014/16.

### Data Collection

Eligible participants attended a designated recruitment site where trained personnel administered questionnaires, including the WHO StepWise questionnaire, (21) and collected clinical and biochemical data. Anthropometry was measured using standardized techniques as recommended by the WHO (21). WC and hip circumference measurements were taken thrice, using a non-elastic measuring tape as per WHO guidelines (21), and averaged for the purposes of this study. Height was measured to the nearest centimeter using a stadiometer. Blood pressure was taken three times at 3-min intervals (21), of which the lowest systolic blood pressure (SBP) and corresponding diastolic blood pressure (DBP) readings were used for analysis.

A 75-g oral glucose tolerance test (OGTT) was administered to determine diabetes mellitus in participants without a history of the condition while known diabetes was self-reported as being previously diagnosed by a doctor (22). Biochemical analyses, using fasting samples, were conducted at an ISO 15189 accredited Pathology practice (PathCare, Reference Laboratory, Cape Town, South Africa). These included plasma glucose; plasma insulin, high-density lipoprotein cholesterol (HDL-C); low-density lipoprotein cholesterol (LDL-C); total cholesterol (TC); triglycerides; ultrasensitive C-reactive protein (CRP); and serum creatinine.

### Definitions

Level of education was categorized as ≤ 7 years of education (up to completion of primary school) and > 7 years of education (secondary schooling and higher). Current tobacco use was objectively defined as cotinine levels >10 ng/mL (23, 24) while alcohol consumption was self-reported Diabetes was defined as fasting plasma glucose ≥ 7.0 mmol/l and/or a 2-h post-OGTT plasma glucose ≥ 11.1 mmol/l or self-reported and/or on diabetes medication. Pre-diabetes: fasting plasma glucose between 6.1 and 6.9 mmol/l or/and a post 2-h glucose between 7.8 mmol/l and 11.1 mmol/l (22). BMI was calculated as weight in kilograms (kg) divided by height in meter squared (m^2^) and classified as normal weight (18.5–24.9 kg/m^2^), overweight (25.0–29.99 kg/m^2^) and obesity (≥ 30.0 kg/m^2^).

Although obesity phenotype and metabolic syndrome share similar characteristics, the two have different approaches in understanding the clustering of cardiometabolic risk factors. Metabolic syndrome has predefined set criteria, with the most recent being the harmonized Joint Interim Statement (JIS) definition (25). In contrast, obesity phenotype currently has no uniform criteria. Per the population being studied, investigators take into consideration a number of cardiometabolic risk factors to categorize participants as “metabolically healthy” or “metabolically abnormal” with further categorization based on obesity status or phenotype (26–29). Obesity phenotypes were defined using body size in combination with certain cardiometabolic risk factors to determine the different categories. For the purpose of this study, the presence of two or more metabolic abnormalities classified participants as metabolically abnormal, while those with one or no metabolic abnormality were considered metabolically health.

The cardiometabolic risk factors used to categorize metabolic status were as follows: (1) SBP ≥ 140 mmHg and/or DBP ≥ 90 mmHg or on blood pressure lowering medications; (2) elevated triglycerides (>1.70 mmol/L); (3) low HDL-C (men: <1.0 mmol/L, women: <1.3 mmol/L); (4) high fasting blood glucose level (>5.5 mmol/L) or on hypoglycaemic agents; and (5) insulin resistance diagnosed using the Homeostatic Model Assessment of Insulin Resistance (HOMA-IR) values above the 90th percentile. In secondary analyses we included a sixth metabolic abnormality: elevated CRP (≥ 3 mg/L).

The BMI and metabolic categories were cross-classified into six obesity sub-phenotypes: (1) normal weight and metabolically healthy (NWMH), (2) normal weight and metabolically abnormal (NWMA), (3) overweight and metabolically healthy (OvMH), (4) overweight and metabolically abnormal (OvMA), (5) obese and metabolically healthy (MHO), and (6) obese and metabolically abnormal (MAO).

### Statistical Analysis

The software's, Statistica v.13 (TIBCO Software Inc. (2017) and SPSS v.25 (IBM Corp, 2011) were used for data analyses. The data was tested for normality using Normal Q-Q Plots. The results for continuous variables are reported as means and standard deviations when normally distributed, medians and 25th and 75th percentiles for skewed variable; while categorical variables are reported as counts and percentages with numbers. For group comparisons, analysis of variance test (ANOVA) and Kruskal Wallis test were used for continuous variables, while the chi-square test was used for categorical variables. The Linear-by-Linear Association (LLA) test (categorical), and Brown-Forsythe Levene test (continuous) were used for linear trends to test the change in metabolic phenotype distribution between the two cross-sectional studies. Two-way and three-way interactions between year of study, BMI and metabolic status in the distribution of participants' characteristics were tested in linear (continuous variables) and logistic (categorical variables) regressions by including in the same model the main effects of the variables to be tested as well as their interaction terms. A *p*-value <0.05 was used to characterize statistically significant results.

### Ethical Approval

Ethical approval for this study was obtained from the University of Cape Town Human Research Ethics Committee (ref: 442/2016) and the Cape Peninsula University of Technology for the 2008/09 and 2014/16 studies (ref. no. CPUT/HW-REC 2008/002 and CPUT/HW-REC 2015/H01). Permission to conduct the surveys was granted by the city and community management. All participants provided written informed consent in accordance with the Declaration of Helsinki.

## Results

### General Characteristics of the Participants

The general characteristics of participants by survey year are summarized in Table 1. Participants in 2014/16 compared to 2008/09 were younger (49.6 ± 15.2 vs. 54.3 ± 14.7 years, *p* < 0.001), slightly better educated (secondary or higher: 65.7 vs. 64.8%, *p* < 0.001) and had lower BMI levels (29.2 ± 8.0 vs. 29.9 ± 7.3 kg/m^2^, *p* < 0.031). In 2014/16 vs. 2008/09, participants also had better glycaemic variables i.e. lower levels of fasting glucose [5.0 (range: 4.6–5.6) vs. 5.6 (range: 5.0–6.5)], 2-h glucose [6.1 (range: 4.9–7.6) vs. 6.8 (range: 5.7–8.7)], HbA1c [6.1 (range: 4.9–7.6) vs. 6.8 (range: 5.7–8.7)] and HOMA-IR [5.8 (range: 5.4–6.2) vs. 5.9 (range: 5.5–6.3)] (all *p* < 0.043), and lower LDL-C levels (3.3 ± 1.0 mmol/l vs. 3.7 ± 1.0 mmol/l), all *p* ≤ 0.043. However, participants in 2014/16 compared with 2008/09 had higher levels of blood pressure (SBP: 136.0 ± 25.9 vs. 123.5 ± 19.2 mmHg, DBP: 85.8 ± 14.9 vs. 74.9 ± 12.5 mmHg, both <0.001) and CRP (4.1 ± mg/L vs. 3.6 ± mg/L, *p* < 0.001).

**Table 1:** Comparison of the characteristics in 2008/09 and 2014/16 by gender.

**Characteristics**	**2008/09**			**2014/16**			**Overall 2008/09 vs. 2014/16**	**F vs. M 2008/09 *p*-value**	**F vs. M 2014/16 *p*-value**	**Women: 2008/09 vs. 2014/16 *p*-value**	**Men: 2008/09 vs. 2014/16 *p*-value**
	**Overall (*n* = 927)** **Mean (SD)**	**Women (*n* = 709)** **Mean (SD)**	**Men (*n* = 220)** **Mean (SD)**	**Overall (*n* = 1969)** **Mean (SD)**	**Women (*n* = 1488)** **Mean (SD)**	**Men (*n* = 481)** **Mean (SD)**					
Age, years	54.3 (14.7)	53.7 (14.4)	56.3 (15.2)	49.6 (15.2)	50.3 (14.9)	47.4 (15.7)	<0.001	0.022	<0.001	<0.001	<0.001
Waist circumference (cm)	97.1 (14.9)	98.0 (14.7)	94.1 (15.1)	92.6 (17.1)	94.7 (16.7)	86.1 (16.6)	<0.001	0.001	<0.001	<0.001	<0.001
Hip circumference (cm)	109.8 (14.8)	112.7 (14.9)	100.3 (10.0)	104.5 (16.4)	107.7 (16.2)	94.5 (12.5)	<0.001	<0.001	<0.001	<0.001	<0.001
Waist-to-hip ratio	0.9 (0.1)	0.9 (0.1)	0.9 (0.1)	0.9 (0.1)	0.9 (0.1)	0.9 (0.1)	0.868	0.169	0.051	0.266	0.384
Systolic blood pressure (mmHg)	123.5 (19.2)	122.5 (19.5)	126.6 (18.0)	136.0 (25.9)	136.3 (25.7)	135.0 (26.5)	<0.001	0.007	0.32	<0.001	0.925
Diastolic blood pressure (mmHg)	74.9 (12.5)	74.5 (12.7)	76.3 (11.7)	85.2 (14.9)	85.8 (14.5)	83.5 (15.8)	<0.001	0.068	<0.001	<0.001	<0.001
Fasting glucose (mmol/L)^*^	5.6 (5.0–6.5)	5.6 (5–6.5)	5.5 (5.0–6.4)	5.0 (4.6–5.6)	5.0 (4.6–5.7)	4.8 (4.4–5.5)	<0.001	0.449	<0.001	<0.001	<0.001
2-h glucose (mmol/L)^*^	6.8 (5.7–8.7)	7.0 (5.7–8.9)	6.4 (5.3–8.4)	6.1 (4.9–7.6)	6.3 (5.2–7.9)	5.1 (4.1–6.7)	<0.001	0.010	<0.001	<0.001	<0.001
HBA1c (%)^*^	5.9 (5.5–6.3)	5.9 (5.5–6.3)	5.9 (5.5–6.3)	5.8 (5.4–6.2)	5.8 (5.5–6.3)	5.6 (5.3–6.0)	0.005	0.847	<0.001	0.073	<0.001
Fasting insulin (mIU/L)^*^	6.8 (2.7–12.8)	7.5 (3.3–13.5)	4.3 (1.8–9.2)	6.8 (4.3–11.2)	7.4 (4.9–11.7)	4.9 (2.9–8.6)	0.414	<0.001	<0.001	0.057	0.023
2-h insulin (mIU/L)^*^	38.1 (19.5–71.6)	42 (22.2–79.6)	25.9 (10.2–45.7)	38.8 (20.7–71.9)	45.3 (25.3–79.6)	21.8 (10.2–43.4)	0.473	<0.001	<0.001	0.096	0.595
HOMA-IR^*^	1.7 (0.7–3.4)	1.9 (0.8–3.7)	1.2 (0.4–2.7)	1.6 (0.9–2.8)	1.8 (1.1–3.1)	1.1 (0.6–2.1)	0.040	<0.001	<0.001	0.623	0.449
Total cholesterol (mmol/L)	5.6 (1.2)	5.2 (1.2)	5.7 (1.2)	5.3 (1.2)	5.3 (1.1)	4.8 (1.1)	0.22	0.134	0.765	0.076	0.835
HDL-cholesterol (mmol/L)	1.3 (0.3)	1.3 (0.4)	1.3 (0.3)	1.4 (0.4)	1.2 (0.3)	1.3 (0.4)	0.183	0.837	0.955	0.168	0.595
LDL-cholesterol (mmol/L)	3.7 (1.0)	3.2 (1.0)	3.8 (1.0)	3.3 (1.0)	3.5 (1.0)	2.9 (1.0)	0.043	0.757	0.601	0.113	0.123
Triglycerides (mmol/L)^*^	1.3 (0.9–1.8)	1.3 (0.9–1.8)	1.3 (0.9–1.8)	1.2 (0.9–1.7)	1.2 (0.90–1.7)	1.1 (0.9–1.7)	<0.001	0.533	0.236	0.228	0.045
C-reactive protein (mg/L)^*^	3.6 (1.0–9.4)	4.0 (1.1–9.9)	2.9 (0.8–7.7)	4.1 (1.7–9.1)	4.5 (1.9–9.9)	2.7 (1.2–6.3)	<0.001	0.023	<0.001	0.007	0.982
Creatinine (umol/L)^*^	81 (71–92)	78 (69–88)	92 (82–104)	59 (52–69)	56 (50–64)	70 (63–82)	<0.001	<0.001	<0.001	<0.001	<0.001
Body mass index (kg/m^2^)	29.9 (7.3)	31.0 (7.2)	26.1 (6.2)	29.2 (8.0)	30.7 (7.9)	24.6 (6.4)	0.031	<0.001	<0.001	0.349	0.003
**Body Mass Index Status**							0.001	<0.001	<0.001	0.067	0.003
Normal (18.5–24.9 kg/m^2^)	27.4 (251/917)	21.3 (149/701)	47.2 (102/216)	34.2 (669/1958)	25.5 (378/1480)	60.9 (291/478)					
Overweight (25 to 29.9 kg/m^2^)	27.4 (251/917)	26.5 (186/701)	30.1 (65/216)	23.6 (462/1958)	23.6 (350/1480)	23.4 (112/478)					
Obese(≥30 kg/m^2^)	45.3 (415/917)	52.2 (366/701)	22.7 (49/216)	42.2 (827/1958)	50.8 (752/1480)	15.7 (75/478)					
**Education level**							0.612	0.056	0.007	0.653	0.856
≤ 7 years, % *(n)*	35.2 (324/920)	36.9 (259/702)	29.8 (65/218)	34.3 (670/1956)	35.9 (531/1479)	29.1 (139/477)					
>7years, % *(n)*	64.8 (596/920)	63.1 (443/702)	70.2 (153/218)	65.7 (1286/1956)	64.1 (948/1479)	70.9 (338/477)					
**Alcohol use**							0.651	<0.001	<0.001	<0.001	<0.001
Currently drinking % *(n)*	27.3 (250/915)	21.2 (148/698)	47 (102/217)	26.5 (519/1957)	22.3 (330/1479)	39.5 (189/478)					
Non-drinker, % *(n)*	72.7 (665/915)	78.8 (550/698)	53 (115/217)	73.5 (1438/1957)	77.7 (1149/1479)	60.5 (289/478)					
**Tobacco use**							0.612	0.074	<0.001	0.019	0.001
Non-smoker, % *(n)*	57.5 (531/923)	59.1 (417/705)	52.3 (114/218)	50.2 (959/1909)	53.8 (776/1442)	39.2 (183/467)					
Smoker, % *(n)*	42.5 (392/923)	40.9 (288/705)	47.7 (104/218)	49.8 (950/1909)	46.2 (666/1442)	60.8 (284/467)					

* *Median (25th−75th percentiles); SD, standard deviation; F, women; M, men; vs, versus; HbA1c, Hemoglobin A1c-; HOMA-IR, Homeostatic Model Assessment of Insulin Resistance; HDL, high density lipoprotein; LDL, low density lipoprotein*.

### Distribution of Body Size phenotypes

**Figure 1:**
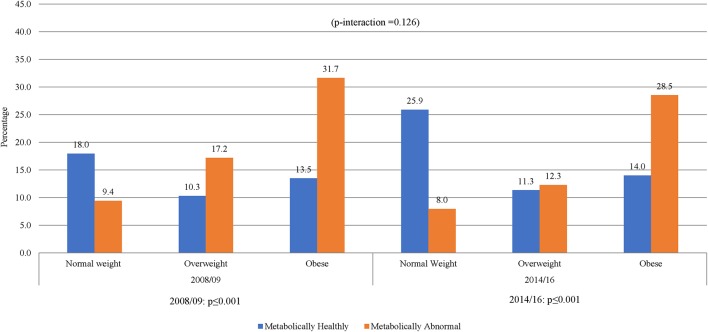
Distribution of obesity phenotypes per year of study. When taking 5 variables into account, NWMH had decreased from 18 to 13.5% MHO. Similar results were shown in 2014/16, NWMH decreasing from 25.9 to 14.0% in MHO, with a non-significant p-interaction between 2008/09 and 2014/16.

**Figure 2:**
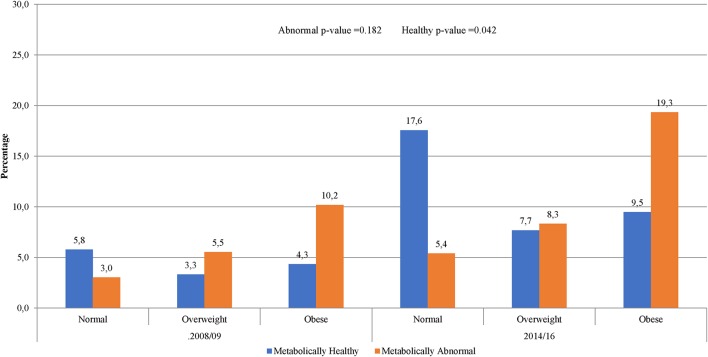
Prevalence metabolic phenotype within BMI categories by year of study.

The prevalence of normal weight, overweight and obesity was, respectively, 27.4, 27.4, and 45.3% in 2008/09 and 34.2, 23.6, and 42.2% in 2014/16; *p* = 0.001 for the difference in the distribution across years (Table 1). A similar pattern was observed in men and women. In 2008/09, 13.5% of the participants were obese yet metabolically healthy (0 or 1 metabolic abnormality present) while 9.4% had normal weight but were metabolically abnormal (2 or more metabolic abnormalities present) (Figure 1). The distribution of obesity phenotypes in 2014/16 was 25.9% (NWMH), 8.0% (NWMA), 11.3% (OvMH), 12.3% (OvMA), 14.0% (MHO), and 28.5% (MAO). There was no difference in the pattern of distribution of obesity phenotype across the two time-points (interaction *p* = 0.126; Figure 1). In both 2008/09 and 2014/16, the proportion of metabolically unhealthy participants steadily increased across BMI categories (both *p* < 0.001 for linear trends; Figure 1). Within BMI categories, the proportion of metabolically abnormal participants (2008/09 vs. 2014/16) was 3.0 vs. 5.4% among the normal weight (*p* = 0.001), 5.5 vs. 8.3% among the overweight (*p* = 0.007), and 10.2 vs. 19.3% among the obese (*p* = 0.276) (Figure 2).

**Figure 3:**
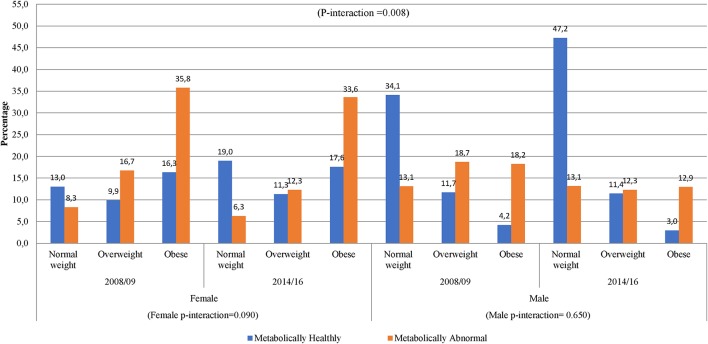
Distribution of obesity phenotypes per year of study and gender. In 2008/09, women had shown an increase from NWMH, 13.0–16.3% in MHO, while in 2014/16 there was a slight decrease from 19.0% in NWMH to 17.6% in MHO (women p-interaction = 0.090). Men had shown a decrease of 8-fold in NWMH to MHO in 2008/09, while 2014/16 had shown a 15-fold decrease (men p-interaction = 0.650).

In women, across the two time points, there was an increase in metabolic abnormalities by BMI category (Figure 3). In men, however, the proportion of metabolic abnormalities was similar across BMI categories in 2014/16 and in overweight and obese in 2008/09. The proportion of metabolically healthy men substantially decreased across increasing BMI categories across both time-points. Interestingly, 34.1% of men in 2008/09 and 47.2% in 2014/16 had normal weight and were metabolically normal. Accordingly, there was a significant gender interaction effect in the distribution of obesity phenotypes across years (interaction *p* = 0.008) (Figure 3).

In secondary analysis accounting for high CRP as an additional metabolic abnormality, the proportion of metabolically abnormal participants linearly increased across rising BMI categories while that for metabolically healthy individuals decreased across both time-points (both *p* < 0.001 for linear trend), with no evidence of interaction by year (interaction *p* = 0.225) (Supplementary Figure 1). As expected, the proportion of metabolically healthy participants decreased overall and across all BMI categories and years of survey. The above patterns were mostly similar in women, but not in men where the proportion of metabolic abnormalities varied less across BMI categories in 2008/09 while a decreasing trend was observed in 2014/16. This apparent different pattern did not result in significant gender^*^year interaction in the distribution of obesity phenotypes (interaction *p* = 0.869) (Supplementary Figure 2).

### The Metabolic Profile Within and Across BMI Categories

Within BMI categories, metabolically abnormal participants compared with their metabolically healthy counterparts, were older (normal weight: 56.0 vs. 44.1 years; overweight: 56.0 vs. 47.4 years and obese 55.1 vs. 50.0 years) (all *p* ≤ 0.035), less educated (all *p* < 0.001) and included fewer drinkers among normal weight (31.9 vs. 42.9%; *p* = 0.003) and obese participants (15.1 vs. 20.2%; *p* = 0.026) (Table 2). However, the proportions of smokers were similar (all *p* > 0.289) as were the proportions of men among normal weight (37.7 vs. 44.7%, *p* = 0.059) and overweight (24.9 vs. 25.3% (*p* = 0.907) metabolically abnormal vs. metabolically healthy participants, respectively. Among obese participants, a higher proportion of men were metabolically abnormal than metabolically healthy (11.9 vs. 5.9%, *p* = 0.001). Unsurprisingly, metabolically abnormal participants compared with metabolically healthy participants within BMI categories displayed significantly worse metabolic profiles. However, some exceptions were WC among all BMI categories (all *p* ≥ 0.070), SBP among overweight and obese participants (both *p* > 0.085), DBP among overweight (*p* = 0.381), TC and LDL-C among overweight and obese (all *p* > 0.102) and HDL-C among normal weight and overweight participants (both *p* > 0.072).

**Table 2:** Pairwise comparison of metabolically healthy and abnormal participants by body mass index.

**Characteristics**	**Normal weight**			**Overweight**			**Obese**			**P trend across BMI categories**			**P interaction**		
	**Healthy** **Mean(SD)**	**Abnormal** **Mean(SD)**	***p***	**Healthy** **Mean(SD)**	**Abnormal** **Mean(SD)**	***p***	**Healthy** **Mean(SD)**	**Abnormal** **Mean(SD)**	***p***	**Overall**	**Healthy**	**Abnormal**	B^*^M	M^*^Y	B^*^M^*^ Y
Prevalence,% *(n)*	23.4 (662)	8.4 (239)		11.0 (312)	13.9 (393)		13.8 (392)	29.5 (837)		<0.001			<0.001		
**Gender**
Women	55.3 (366/662)	62.3 (149/239)	0.059	74.7 (233/312)	75.1 (295/393)	0.907	94.1 (369/392)	88.1 (737/837)	0.001	<0.001	<0.001	<0.001	0.001	0.449	0.335
Men	44.7 (296/662)	37.7 (90/239)		25.3 (79/312)	24.9 (98/393)		5.9 (23/392)	11.9 (100/837)							
Age, years	44.1 (16)	56.0 (15)	0.035	47.4 (15.3)	56.0 (13.7)	0.008	50.0 (14.5)	55.1 (12.6)	<0.001	<0.001	<0.001	0.407	<0.001	0.550	<0.001
Waist circumference	76.1 (8.7)	81.1 (7.9)	0.892	89.8 (8.7)	94 (7.5)	0.070	103.9 (11.5)	109.1 (11.9)	0.479	<0.001	<0.001	<0.001	0.559	0.001	0.084
Hip circumference	90.6 (8.7)	92.3 (7.1)	0.021	102.8 (7.0)	102.8 (6.1)	0.028	118.6 (12.0)	119.8 (13.1)	0.037	<0.001	<0.001	<0.001	0.271	<0.001	0.113
Waist-to-hip ratio	0.8 (0.1)	0.9 (0.1)	0.150	0.9 (0.1)	0.9 (0.1)	0.031	0.9 (0.1)	0.9 (0.1)	0.639	<0.001	<0.001	<0.001	0.875	0.959	0.853
Systolic blood pressure	116.0 (20.4)	136.2 (25.5)	<0.001	120.4 (20.9)	132.1 (22.5)	0.085	122.9 (20.4)	133.0 (21.5)	0.160	<0.001	<0.001	0.076	<0.001	<0.001	<0.001
Diastolic blood pressure	74.7 (13.3)	83.1 (17.1)	<0.001	77.4 (13.0)	80.8 (12.7)	0.381	79.0 (12.3)	83.6 (13.5)	0.041	<0.001	<0.001	0.005	0.001	0.001	<0.001
Fasting glucose ^*^	4.7 (4.3–5)	5.6 (4.8–6.4)	<0.001	4.9 (4.5–5.2)	5.8 (5.1–7.9)	<0.001	5.0 (4.6–5.2)	5.9 (5.1–7.5)	<0.001	<0.001	<0.001	0.003	0.045	0.253	0.267
2-h glucose^*^	5.2 (4.3–6.4)	6.4 (5.3–8.2)	<0.001	5.7 (4.8–6.7)	7.1 (5.8–9.1)	<0.001	6.2 (5.4–7.5)	7.6 (6.4–9.8)	<0.001	<0.001	<0.001	<0.001	0.178	0.037	0.099
HBA1c^*^	5.5 (5.2–5.7)	5.8 (5.4–6.2)	<0.001	5.6 (5.4–5.9)	6.1 (5.7–7.4)	<0.001	5.7 (5.5–6)	6.2 (5.8–7.1)	<0.001	<0.001	<0.001	<0.001	0.012	0.023	0.054
Fasting insulin^*^	3.5 (2.4–5.4)	5.2 (2.9–8.3)	<0.001	5.9 (4–8.6)	7.6 (5.4–11.6)	<0.001	7.6 (5.3–11.7)	11.7 (7.3–17.6)	<0.001	<0.001	<0.001	<0.001	0.024	0.531	0.16
2-h insulin ^*^	21.2 (11–35.4)	28.6 (14.7–47.6)	<0.001	31.0 (18.3–50.5)	49.1 (28.7–83.8)	<0.001	50.8 (27.3–83)	67.1 (40.6–112.5)	<0.001	<0.001	<0.001	<0.001	0.021	0.637	0.145
HOMA-IR^*^	0.8 (0.5–1.2)	1.3 (0.7–2.2)	<0.001	1.3 (0.9–1.9)	2.2 (1.4–3.7)	<0.001	1.7 (1.2–2.6)	3.2 (1.9–5.5)	<0.001	<0.001	<0.001	<0.001	0.007	0.962	0.121
Total cholesterol	4.9 (1.1)	5.4 (1.2)	0.042	5.4 (1.2)	5.7 (1.3)	0.102	5.4 (1.1)	5.5 (1.2)	0.111	<0.001	<0.001	0.009	0.009	0.29	0.053
HDL-cholesterol	1.5 (0.4)	1.3 (0.4)	0.451	1.4 (0.3)	1.2 (0.3)	0.072	1.4 (0.3)	1.2 (0.3)	<0.001	<0.001	<0.001	<0.001	0.986	0.687	0.994
LDL-cholesterol	2.9 (0.9)	3.3 (1.1)	0.007	3.5 (1.0)	3.6 (1.0)	0.629	3.5 (1.0)	3.5 (1.0)	0.185	<0.001	<0.001	0.018	0.001	0.755	0.008
Triglycerides^*^	0.9 (0.7–1.1)	1.5 (1–2)	<0.001	1.0 (0.8–1.3)	1.8 (1.3–2.3)	<0.001	1.1 (0.8–1.3)	1.7 (1.2–2.2)	<0.001	<0.001	<0.001	0.003	0.257	0.013	0.099
C-reactive protein ^*^	1.8 (0.7–5.1)	2.5 (0.9–5)	0.041	2.5 (1–5)	3.3 (1.5–7.7)	<0.001	5.6 (2.5–10.3)	7.2 (3.8–13.5)	<0.001	<0.001	0.002	<0.001	0.002	0.338	0.004
Creatinine^*^	63 (54–75)	68 (59–86)	<0.001	65.5 (55–80)	69 (56–87)	0.026	62 (53–74)	66 (55–82)	0.002	0.033	0.014	0.51	0.513	0.612	0.781
**Education level**			<0.001			<0.001			<0.001	0.195	0.627	0.105	0.07	0.401	0.855
≤ 7 years	27.2 (179/658)	47.3 (112/237)		26.1 (80/307)	40.8 (160/392)		29.2 (114/390)	39.6 (329/831)							
>7years	72.8 (479/658)	52.7 (125/237)		73.9 (227/307)	59.2 (232/392)		70.8 (276/390)	60.4 (502/831)							
**Alcohol use**			0.003			0.454			0.026	<0.001	<0.001	<0.001	0.613	0.555	0.785
Non drinker	57.1 (375/657)	68.1 (162/238)		72.2 (223/309)	74.7 (292/391)		79.8 (312/391)	84.9 (702/827)							
Currently drinking	42.9 (282/657)	31.9 (76/238)		27.8 (86/309)	25.3 (99/391)		20.2 (79/391)	15.1 (125/827)							
**Tobacco use**			0.297			0.289			0.515	<0.001	<0.001	<0.001	0.179	0.048	0.348
Non-smoker	26.4 (171/647)	30 (71/237)		54.5 (169/310)	58.5 (223/381)		67.3 (261/388)	69.1 (571/826)							
Smoker	73.6 (476/647)	70 (166/237)		45.5 (141/310)	41.5 (158/381)		32.7 (127/388)	30.9 (255/826)							

Values are percentage, %(count), SD, standard deviation;

* *median (25th−75th percentile). B vs. M, interaction term of body mass index categories and metabolic status; M vs. Y, metabolic status and year of study; B vs. M vs. Y, body mass index categories, metabolic status and year of study. Units of measurements and other conventions are as per Table 1*.

Across BMI categories, the proportion of men decreased from 44.8% in NWMH to 5.9% in MHO, and from 37.7% in NWMA to 11.9% in MAO (both *p* < 0.001 for linear trend). Age linearly increased across rising BMI categories, driven by a steeper trend among metabolically health participants (*p* < 0.001), and only a flat pattern among metabolically abnormal participants (*p* = 0.407). Across the BMI categories, current drinking, WC, DBP, hip circumference, fasting glucose, 2 h glucose, HbA1c, fasting insulin, 2 h insulin, TC, LDL-C, triglycerides and CRP linearly increased in both metabolic healthy and abnormal participants (all *p* ≤ 0.018 for linear trends). SBP significantly increased across rising BMI category among metabolically health participants only (*p* < 0.001 for linear trend); while current smoking linearly decreased in both the metabolic healthy and abnormal participants (both, *p* < 0.001 for linear trend). No linear trend was observed for education level across increasing BMI category both in metabolically healthy and abnormal participants (all *p* ≥ 0.105 for linear trends) (Table 2).

In secondary analysis accounting for high CRP as an additional metabolic abnormality, despite some attenuation of some *p*-values, the overall pattern was broadly the same (Supplementary Table 1).

### Interaction by BMI Category, Metabolic Profile, and Year of Study

Significant interactions between BMI categories and metabolic status were apparent in the distribution of gender (interaction *p* = 0.001), age (*p* < 0.001), SBP and DBP (both *p* < 0.001), fasting glucose (*p* = 0.045), HbA1c (*p* = 0.012), fasting insulin (*p* = 0.024), 2 h insulin (*p* = 0.021), HOMA-IR (*p* = 0.007), TC (*p* = 0.009), and CRP (*p* = 0.002) (Table 2). Furthermore, significant interactions between metabolic status and year of study (2008/09 vs. 2014/16) were apparent in the distribution of WC, hip circumference, SBP, DBP, 2 h glucose, HbA1c, triglycerides and tobacco use (all interactions *p* ≤ 0.048). Significant 3-way interactions BMI categories^*^metabolic status^*^ year of study were found in the distribution of age, SBP, DBP, LDL-C, and CRP (all 3-way interaction *p* ≤ 0.008). These were indications that some of the variations in participants' profiles across BMI categories and metabolic status were occurring in differential ways across the two years of study.

## Discussion

In the current study, there was a differential distribution of BMI status across the two time-points, reflecting fewer overweight and more normal-weight participants in 2014/16 compared to 2008/09. However, the distribution of obesity phenotypes (that is the combination of BMI categories and underlying metabolic burden) did not differ by year of study, with about one in ten participants having normal weight and being metabolically abnormal and 13–14% being obese and metabolically healthy. Findings were also broadly consistent, regardless of whether or not subclinical inflammation (high CRP levels) was accounted for as a metabolic abnormality. Variable differences were apparent in the distribution of cardiometabolic risk profile and other key characteristics across BMI categories and metabolic status, with suggestions that some of these variations were occurring in differential ways across the 2 years of the study.

In 2016, the Government of South Africa issued a strategy for the prevention and control of obesity 2015–2016 with the target to have no increase in 2016, 3% reduction in 2017 and 10% reduction in 2020 (30). Even if effective, this policy issued when our second survey was underway, would not have accounted for differences, if any (or the lack thereof), in obesity phenotype distribution between the two time points of our study. The distribution of BMI status changed across the two time points, driven by changes in the distribution in men, but not in women. The changes in men could be attributed to their younger age in 2014/16 compared with 2008/09. This was shown in the 2016 South African Demographic and Health Survey, where young participants had a lower prevalence of obesity (31).

A number of previous studies in developed countries have investigated obesity phenotypes (27, 32). In Africa, however, such studies (26, 28) are few, with even fewer done in the mixed ancestry population in South Africa (33). In contrast to the findings in urban Cameroonians, where a small proportion of normal weight participants were metabolically abnormal (0.3%), a considerable percentage of NWMA participants were found in this study (8.4%), in line with a similar a study in the HIV-infected population in Cape Town (28). A recent meta-analysis of 40 studies globally reported that over one-third of the obese population was metabolically healthy (34). This is similar to our findings in both surveys, but varied considerably from studies in Cameroonians and Brazilians where higher rates of MHO were reported (26, 34). These differences have been ascribed to different ages, sample sizes and criteria used to define obesity phenotypes across studies (9, 27, 32, 35–38).

In our study, findings in women were broadly similar to those observed in the overall sample, reflecting the high proportion of women in this study. However, in the analyses stratified by gender, differences between men and women were apparent. Metabolically healthy but obese phenotype has been reported to occur more frequently in adult women compared to men (39) as was demonstrated in this study. Indeed, more than 50% of women were obese, but a significant number of them were metabolically healthy (33%), whereas more than 80% of men who were obese were metabolically abnormal. The differences in obesity phenotypes between men and women have been attributed to body shape and fat distribution (40). Women are known to have more subcutaneous adipose tissue than men, especially in the abdominal and gluteo-femoral regions (41, 42), as well as lower waist-to-hip ratios, which was also observed in this study. Lower waist-to-hip ratio is indicative of a comparatively more gynoid shaped which is associated with less metabolic complications (18, 41, 43). MHO women, tended to be younger and smoked and drank moderately compared to men of all ages (44–46). In our study, however, there were no differences in smoking and alcohol consumption between the MHO and MAO. Furthermore, MHO rates in men were similar between the two surveys despite a significant difference in age between the two studies.

The distribution of cardiometabolic risk factors differed across BMI categories and obesity phenotype, which was similar to previous reports involving different population groups (26, 28, 29, 44, 47, 48). For example, in a study by Pajunen et al. (29) Caraballo et al. (23) conducted in the Finnish population, significant interactions between phenotype and BMI were found in the distribution of fasting blood glucose, fasting insulin and HOMA-IR, in keeping with our findings.

The major strength of this study is that the two cross-sectional surveys analyzed were conducted in the same population, using similar methodological approaches. The limitations of this study are as follows: [1] the surveys were conducted in only one population group in Cape Town; thus, findings cannot be easily extrapolated to other ethnic groups, [2] comparisons in the current analyses were based on only two cross-sectional surveys which prevented a reliable assessment of time-trends, [3] the proportion of men included in these surveys was small; consequently, the overall study findings reflect more the observations in women. In general, population-based surveys in South Africa have reported a low participation of men (49).

## Conclusion

In conclusion, our study has shown negligible changes in obesity phenotype distribution between 2008/09 and 2014/16 in the South African mixed ancestry population. Furthermore, there was a high burden of metabolic abnormalities among normal weight participants, and a significant proportion of obese individuals who were metabolically healthy. Additional research is needed to improve risk stratification for obesity phenotypes; this may enable targeted screening and cost-effective detection of individuals at high risk for cardiometabolic diseases.

## Data Availability Statement

The datasets generated and/or analyzed during the current study are not publicly available due to the terms of consent to which participants agreed but are available from the principal investigator of the main study on reasonable request.

## Ethics Statement

The studies involving human participants were reviewed and approved by University of Cape Town Human Research Ethics Committee (ref: 442/2016) and Cape Peninsula University of Technology (ref. no. CPUT/HW-REC 2008/002 and CPUT/HW-REC 2015/H01). The patients/participants provided their written informed consent to participate in this study.

## Author Contributions

SD had drafted the article, statistical analysis interpretation of data, and discussion. TM had approved conception was part of the decisions on the analysis and interpretation of data, discussion and had proof read, and corrected the article. NP had approved the conception had given comments on the interpretation of data, discussion and proof reading, and correcting of articles. RE had commented and amended on all aspects of the article, i.e., background, methodology, and discussion. AK had approved conception was part of the decisions on the analysis and interpretation of data, discussion, had proof read, and corrected the article as well as given final approval of the article. All authors had read and approve the final manuscript.

### Conflict of Interest

The authors declare that the research was conducted in the absence of any commercial or financial relationships that could be construed as a potential conflict of interest.

## Abbreviations

WHO: World Health Organization
WC: Waist circumference
SBP: Systolic blood pressure
DBP: Diastolic blood pressure
HDL-C: high density lipoprotein cholesterol
LDL-C: low density lipoprotein cholesterol
TC: total cholesterol
CRP: ultrasensitive C-reactive protein
BMI: Body mass index
HOMA-IR: Homeostatic Model Assessment of Insulin Resistance
NWMH: normal weight and metabolically healthy
NWMA: normal weight and metabolically abnormal
OvMH: overweight and metabolically healthy
OvMA: overweight and metabolically abnormal
MHO: obese and metabolically healthy
MAO: obese and metabolically abnormal.
