# Supplementary material for: Changes in Obesity Phenotype Distribution in Mixed-ancestry South Africans in Cape Town Between 2008/09 and 2014/16
Source: Front Endocrinol (Lausanne). 2019 Nov 6;10:753. doi: 10.3389/fendo.2019.00753 (PMC6851026; doi:10.3389/fendo.2019.00753)
Supplement: Supplementary file 1 [file Data_Sheet_1.pdf]

Supplementary Material

1 Supplementary Figures

Supplementary Figure 1: Distribution of obesity phenotypes per year of study using secondary data analysis, i.e. when adding a sixth variable to determine phenotype, NWMH had shown a decrease from 15.1% in 2008/09 to 6.2% in MHO. As for 2014/16, similar results are shown with NWMH to MHO decreasing from 20.1% to 5.5%.

Supplementary Figure 2: Secondary analysis determining the distribution of obesity phenotypes per year of study and gender. In women there was a similar decreases in both 2008/09 and 2014/16 between NWMH to MHO, p-interaction=0.334. Similarly, in men there was a decreases between NWMH and MHO, with 2008/09, 25.6% to 3.3% and 2014/16, 36.1% to 1.9%, p-interaction=0.710.

2 Supplementary Table

Supplementary Table1: Secondary data pairwise comparison of metabolically healthy and abnormal participants by body mass index (including CRP as a factor)

**Characteristics**	**Normal weight**			**Overweight**			**Obese**			**P trend across BMI categories**			**P interaction**		
	**Healthy Mean (SD)**	**Abnormal Mean (SD)**	**p-value**	**Healthy Mean (SD)**	**Abnormal Mean (SD)**	**p-value**	**Healthy Mean (SD)**	**Abnormal Mean (SD)**	**p-value**	**Overall**	**Healthy**	**Abnormal**	**B*M**	**M*Y**	**B*M*Y**
Prevalence, % (n)	18.5 (524)	13.2 (374)		7.6 (217)	17.2 (489)		5.7 (163)	37.8 (1073)		<0.001					
**Gender**			0.881			0.911			0.921	<0.001	<0.001	<0.001	0.791	0.266	0.211
Women	57.3 (300/524)	57.8 (216/374)		74.7 (162/217)	75.1 (367/489)		90.2 (147/163)	89.9 (965/1073)							
Men	42.7 (224/524)	42.2 (158/374)		25.3 (55/217)	24.9 (122/489)		9.8 (16/163)	10.1 (108/1073)							
Age, years	43.6 (16.1)	52.8 (15.7)	0.250	45.3 (15)	55.2 (14.1)	0.134	51.6 (14.6)	53.7 (13.3)	0.068	<0.001	<0.001	0.033	<0.001	0.368	<0.001
Waist circumference	75.9 (7.9)	79.6 (9.4)	0.006	88.7 (8.7)	93.7 (7.6)	0.150	102.1 (11.5)	108.3 (12)	0.466	<0.001	<0.001	<0.001	0.064	0.006	0.005
Hip circumference	90.9 (8.2)	91.3 (8.5)	0.722	102.5 (7.4)	102.9 (6.1)	0.004	116.9 (12.1)	119.8 (12.9)	0.254	<0.001	<0.001	<0.001	0.042	0.007	0.031
Waist-to-hip ratio	0.8 (0.1)	0.9 (0.1)	0.553	0.9 (0.1)	0.9 (0.1)	0.082	0.9 (0.1)	0.9 (0.1)	0.420	<0.001	<0.001	<0.001	0.332	0.61	0.657
Systolic blood pressure	115.5 (19.2)	129.8 (26.6)	<0.001	118.3 (19)	130.8 (23)	0.001	119.7 (18.2)	131.2 (21.7)	0.009	<0.001	0.020	0.569	0.466	0.017	0.146
Diastolic blood pressure	74.3 (12.2)	80.6 (17.4)	<0.001	76.5 (12.7)	80.6 (12.9)	0.105	77.1 (10.5)	82.8 (13.4)	0.003	<0.001	0.011	0.003	0.332	0.006	0.029
Fasting glucose *	4.6 (4.3-5)	5.0 (4.6-6)	<0.001	4.8 (4.5-5.1)	5.6 (5-7.1)	<0.001	5.0 (4.7-5.2)	5.6 (4.9-6.9)	<0.001	<0.001	0.008	<0.001	0.005	0.019	0.013
2-hour glucose*	5.2 (4.3-6.2)	6.1 (5-8)	<0.001	5.6 (4.8-6.7)	6.6 (5.6-8.6)	<0.001	6.3 (5.4-7.5)	7.2 (6-9)	<0.001	0.001	<0.001	<0.001	0.256	0.009	0.072
HBA1c*	5.5 (5.2-5.7)	5.7 (5.4-6.2)	<0.001	5.6 (5.4-5.8)	6.0 (5.6-7)	<0.001	5.7 (5.5-5.9)	6.1 (5.7-6.7)	<0.001	<0.001	0.001	<0.001	0.001	0.368	0.035
Fasting insulin*	3.6 (2.4-5.5)	4.4 (2.7-7.5)	<0.001	5.7 (3.8-8.7)	7.2 (5.1-10.9)	<0.001	7.5 (5.3-11.1)	10.5 (6.8-16.1)	<0.001	<0.001	<0.001	<0.001	0.068	0.955	0.232
2-hour insulin *	21.2 (10.7-35.8)	25.1 (12.8-43.3)	0.006	31.6 (17.6-50.7)	43.7 (26-77.8)	<0.001	52.2 (27.8-83.8)	62.5 (36.8-106.2)	0.004	<0.001	<0.001	<0.001	0.073	0.993	0.239
HOMA-IR*	0.8 (0.5-1.2)	1.0 (0.6-1.8)	<0.001	1.2 (0.8-1.8)	2.0 (1.2-3.3)	<0.001	1.6 (1.2-2.5)	2.8 (1.6-4.8)	<0.001	<0.001	<0.001	<0.001	0.034	0.578	0.285
Total cholesterol	4.9 (1.1)	5.1 (1.3)	<0.001	5.4 (1.1)	5.6 (1.3)	0.007	5.4 (1)	5.5 (1.2)	0.640	<0.001	<0.001	<0.001	0.474	0.873	0.604
HDL-cholesterol	1.5 (0.4)	1.3 (0.5)	0.360	1.4 (0.4)	1.2 (0.3)	0.036	1.5 (0.3)	1.2 (0.3)	0.187	<0.001	0.007	<0.001	0.661	0.514	0.92
LDL-cholesterol	3.0 (0.9)	3.2 (1.1)	0.003	3.5 (1)	3.6 (1.1)	0.080	3.5 (1)	3.5 (1)	0.095	<0.001	<0.001	<0.001	0.174	0.828	0.426
Triglycerides*	0.9 (0.7-1.1)	1.2 (0.9-1.8)	<0.001	1.0 (0.8-1.3)	1.6 (1.1-2.1)	<0.001	1.1 (0.8-1.3)	1.5 (1.1-2)	0.326	<0.001	<0.001	<0.001	0.056	0.351	0.106
C-reactive protein *	1.2 (0.7-2.5)	4.3 (1.7-9.9)	<0.001	1.5 (0.7-2.7)	4.2 (1.9-8.3)	<0.001	2.1 (1.2-4.4)	7.4 (4.1-13.6)	<0.001	<0.001	0.024	0.202	0.427	0.451	0.66
Creatinine*	64 (55-75.3)	66 (56-83.3)	0.007	65 (55-77.5)	69 (56-86)	0.019	64 (55-78)	64 (54-80)	0.667	0.033	0.253	0.107	0.711	0.728	0.969
**Education level**			<0.001			<0.001			0.055	0.181	0.247	0.115	0.086	0.419	0.521
<=7 years	25.1 (131/522)	43.5 (161/370)		22.1 (47/213)	39.6 (193/487)		29.6 (48/162)	37.4 (399/1066)							
> 7years	74.9 (391/522)	56.5 (209/370)		77.9 (166/213)	60.4 (294/487)		70.4 (114/162)	62.6 (667/1066)							
**Alcohol use**			0.026			0.010			0.888	<0.001	<0.001	<0.001	0.455	0.817	0.666
Non drinker	57.0 (297/521)	64.4 (239/371)		66.8 (143/214)	76.2 (371/487)		82.7 (134/162)	83.2 (884/1063)							
Currently drinking	43.0 (224/521)	35.6 (132/371)		33.2 (71/214)	23.8 (116/487)		17.3 (28/162)	16.8 (179/1063)							
**Tobacco use**			0.389			0.478			0.365	<0.001	<0.001	<0.001	0.239	0.355	0.292
Non-smoker	28.4 (147/517)	25.8 (95/368)		54.9 (118/215)	57.8 (275/476)		71.6 (116/162)	68.1 (718/1055)							
Smoker	71.6 (370/517)	74.2 (273/368)		45.1 (97/215)	42.2 (201/476)		28.4 (46/162)	31.9 (337/1055)							

Values are percentage, % (count), SD-standard deviation, *median (25th-75th percentile). B vs. M, interaction term of body mass index categories and metabolic status; M vs. Y, metabolic status and year of study; B vs. M vs. Y, body mass index categories, metabolic status and year of study. Units of measurements and other conventions are as per table 1.1
