# Supplementary figures and images for: Changes in Obesity Phenotype Distribution in Mixed-ancestry South Africans in Cape Town Between 2008/09 and 2014/16
Source: Front Endocrinol (Lausanne). 2019 Nov 6;10:753. doi: 10.3389/fendo.2019.00753 (PMC6851026; doi:10.3389/fendo.2019.00753)

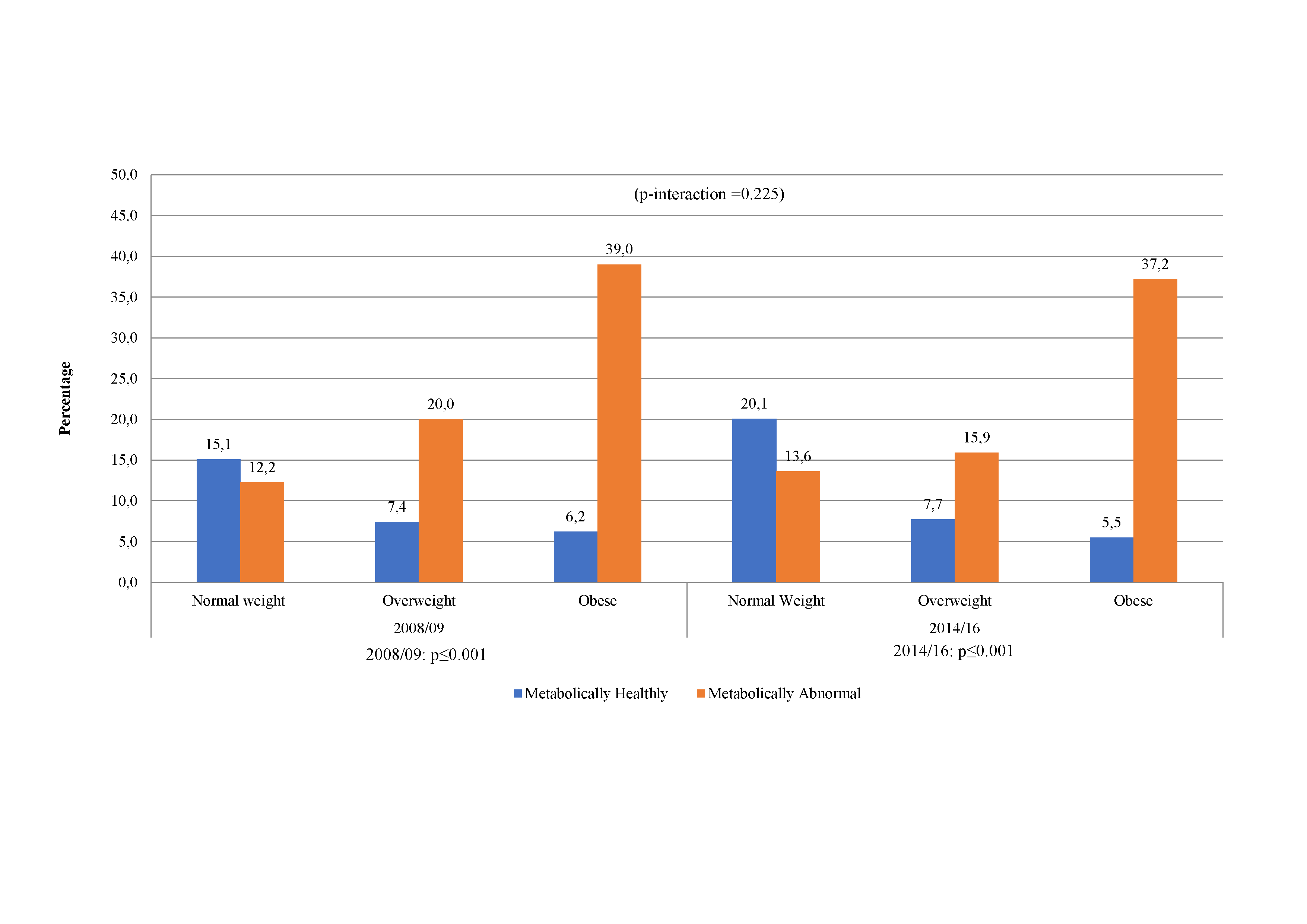

Supplement: Supplementary file 2 [file Image_1.TIFF]

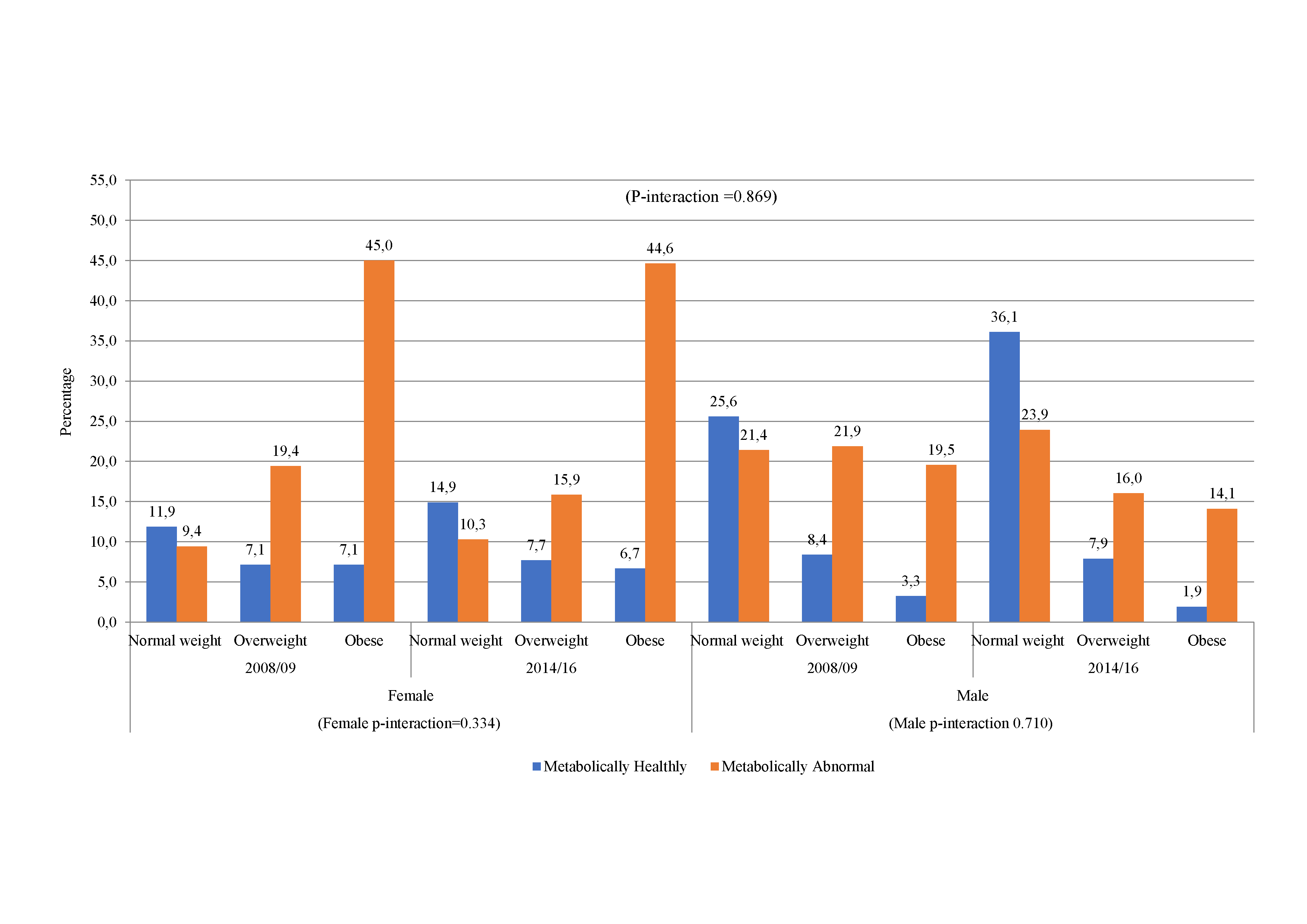

Supplement: Supplementary file 3 [file Image_2.TIFF]
